# Association Between Prudent Dietary Pattern and Cardiometabolic Indicators in a Healthy Qatari Population: Evidence from the Qatar Biobank (QBB)

**DOI:** 10.3390/nu18162607

**Published:** 2026-08-10

**Authors:** Maria M. AlAnazi, Julie A. Lovegrove, Zumin Shi, Karani Santhanakrishnan Vimaleswaran

**Affiliations:** 1 Department of Nutrition Sciences, College of Health Sciences, Qatar University, Doha P.O. Box 2713, Qatar; zumin@qu.edu.qa; 2 Hugh Sinclair Unit of Human Nutrition, Department of Food and Nutritional Sciences, University of Reading, Reading RG6 6DZ, UK; j.a.lovegrove@reading.ac.uk; 3 Institute for Food, Nutrition and Health (IFNH), University of Reading, Reading RG6 6AH, UK

**Keywords:** prudent dietary pattern, cardiometabolic health, body composition, insulin, diastolic blood pressure, Qatar Biobank

## Abstract

**Background/Objectives**: Diet is recognized as an important determinant of cardiometabolic health, yet evidence from Middle Eastern populations remains scarce. This cross-sectional study investigated the association between a Prudent dietary pattern and multiple indicators of obesity, type-2 diabetes, and cardiovascular disease in a metabolically healthy Qatari cohort. **Methods**: Data were obtained from 6919 Qatari adults from the Qatar Biobank study. Participants with diagnosed metabolic disorders, pregnancy, or non-fasting blood samples were excluded. Anthropometric, biochemical, and clinical measurements were collected using standardized protocols. Dietary intake was assessed using a 102-item food frequency questionnaire, and a Prudent dietary pattern was derived through factor loadings analysis. Participants were categorized into low and high intake groups of the Prudent dietary pattern. Associations between the Prudent dietary pattern and cardiometabolic markers were examined using generalized linear models adjusted for age, sex, adiposity, and physical activity with Bonferroni correction applied for multiple testing. **Results**: After correction for multiple testing, greater adherence to the Prudent dietary pattern was significantly associated with lower fat mass index (β = −0.02, *p* = 0.0001), fasting insulin (β = −0.05, *p* = 0.0021), and higher fat-free mass (β = 0.01, *p* = 5.7 × 10^−5^). **Conclusions**: Overall, in a metabolically healthy Qatari population, greater compliance to a Prudent dietary pattern was favorably associated with fat and muscle mass, and insulin regulation after adjusting for age, sex, BMI and physical activity. These findings support the role of dietary habits in greater cardiometabolic health in Middle Eastern Arab populations.

## 1. Introduction

Cardiometabolic diseases (CMDs) encompass a spectrum of interrelated conditions that include metabolic disorders, such as obesity and type 2 diabetes (T2D), along with cardiovascular complications, including ischemic heart disease and heart failure [1]. Collectively, CMDs are among the leading causes of premature mortality and disability worldwide, underscoring their considerable public health burden [2,3], with cardiovascular disease (CVD) remaining the predominant contributor to death [4]. Beyond their health consequences, CMDs, including CVD and diabetes, account for roughly 15% of total health care spending, further highlighting their substantial economic impact [5]. In Qatar, recent evidence underscores the growing burden of CMDs, with obesity affecting approximately 46% of women and 36% of men [6], and type 2 diabetes (T2D) estimated at 17% in the total population in 2021 [7]. Notably, T2D has also been reported in 50–70% of individuals with CVDs [8,9], while CVDs themselves remain the leading cause of mortality, accounting for nearly one-quarter of all deaths nationally [10]. Population-level surveys further indicate that T2D and prediabetes together affect 35–40% of Qatari adults [11], underscoring the urgent need for strategies targeting prevention and early intervention to curb the escalating CMDs burden in the country.

Within this broader context, obesity develops through complex interactions between genetic, biological, environmental, and behavioral determinants [12,13,14]. Lifestyle factors hold particular importance, as their modifiable nature provides a strategic opportunity to attenuate the risk of obesity and its associated comorbidities [15,16]. Diet is one of the most influential of these factors, with extensive evidence linking dietary quality to the prevention of obesity, T2D, and CVD [17,18]. Adherence to healthier dietary patterns has been consistently associated with improvements in lipid metabolism, glycemic control, blood pressure, and systemic inflammation, thereby lowering the risk of T2D and cardiovascular complications [19]. Moreover, such patterns are associated with maintaining favorable metabolic profiles even among individuals with obesity and reducing the risk of progression to metabolically unhealthy states that increase CMD risk [20]. Collectively, these observations emphasize the importance of evaluating overall dietary patterns, which better reflect real-world eating behaviors by capturing the synergistic effects of foods and nutrients, and providing a more comprehensive understanding of diet–disease relationships and health outcomes [21,22].

Rapid globalization, unionization and economic growth have profoundly influenced dietary environments and habits in the Gulf and Qatar. [23,24]. With over 90% of national food supply sourced through imports [24], traditional dietary practices have increasingly been replaced with Westernized energy-dense diets, characterized by high intakes of sugar, meat, refined carbohydrates, and sodium, while fiber-rich foods such as fruits and vegetables remain under-consumed [25,26,27]. Comparable dietary shifts have also been documented across other Gulf Cooperation Council countries, indicating that this is a broader regional challenge rather than one confined to Qatar [28,29,30]. Thus, the rapid pace of modernization has been accompanied by escalating obesity prevalence, which is projected to affect more than half of Qatari adults by 2035, alongside an increased risk of cardiometabolic complications [31,32]. Among healthier options, the Prudent dietary pattern, characterized by higher consumption of vegetables, legumes, whole grains, nuts, seeds, and lean proteins, represents a promising dietary framework with potential protective effects [33,34,35]. Nevertheless, there is limited evidence on how adherence to such patterns may mitigate these risks in Qatar. Given the consistent associations reported between Prudent dietary patterns and improved cardiometabolic health in other populations [36,37,38,39,40,41], this study aimed to evaluate whether similar associations are present in a metabolically healthy Qatari population, where evidence remains limited. We hypothesized that greater adherence to a Prudent dietary pattern would be associated with more favorable obesity, glycemic, lipid, and blood pressure indicators.

## 2. Materials and Methods

### 2.1. Study Participants

**Figure 1:**
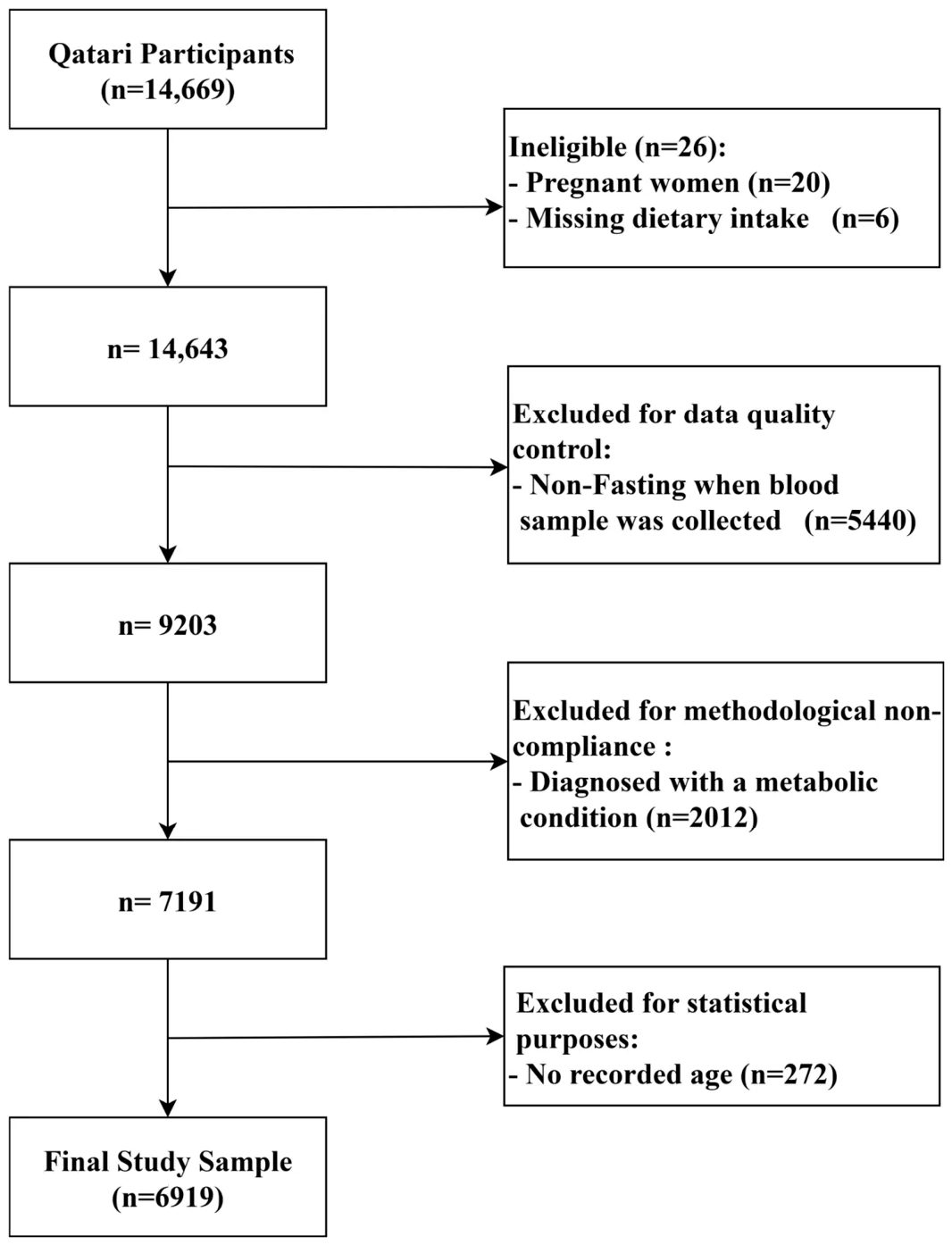
Flowchart of participant selection from the Qatar Biobank dataset. A total of 14,669 Qatari adults were initially considered. After excluding ineligible participants (*n* = 26; 20 pregnant women and 6 with missing dietary intake), 14,643 participants remained. Further exclusions were applied for non-fasting blood samples (*n* = 5440), diagnosed metabolic conditions (*n* = 2012), and missing age data (*n* = 272); the final analytic sample included 6919 individuals.

The dataset was obtained from the Qatar Biobank (QBB), a population-based cohort initiated in 2012 under the Qatar Precision Health Institute (QPHI). The study sought to enroll up to 60,000 participants, including Qatari nationals and long-term residents. Full details of the study design have been described in earlier publications [42,43]. In our cross-sectional investigation, participants with diagnosed metabolic conditions, including T2D, hypertension, hypercholesterolemia, and CVDs, as well as pregnant women, were excluded from the study. The baseline cohort consisted of 14,669 Qataris. Following the removal of individuals with missing dietary intake data (*n* = 6) and reported pregnancy (*n* = 20), the sample was reduced to 14,643. Further exclusions were made for participants who were not fasting during blood collection (*n* = 5440) and those with a self-reported physician diagnosis of a metabolic disorder, including T2D, CVD, hypertension, or hypercholesterolemia (*n* = 2012), as documented in the nurse-administered questionnaire. An additional 272 individuals were excluded due to missing age information, because age was included as a covariate in all analyses. After these steps, the final analytical sample comprised 6919 metabolically healthy Qatari participants (Figure 1). Ethical approval and data access were initially granted by the Qatar Biobank Institutional Review Board (IRB: QF-QBB-RES-ACC-00085-0197) on 19 May 2022, and the dataset was first accessed on 1 June 2022. Two subsequent IRB renewals were obtained, extending the approval period until 18 May 2025. All participant data were fully de-identified prior to access, and no personal identifiers were available to the research team, as each participant was assigned a unique dummy ID. All participants provided informed consent prior to their enrolment in the QBB study [43].

### 2.2. Assessment of Cardiometabolic Phenotypic Markers

The phenotypic markers were selected a priori to represent four major cardiometabolic domains: adiposity and body composition, glycemic regulation, lipid metabolism, and blood pressure. The 14 markers included in these domains were considered the primary study outcomes and were included in the Bonferroni correction for multiple testing. All anthropometric measures were obtained through standardized physical and clinical assessments conducted by trained nursing staff [43]. Obesity-related indicators considered in this study were body mass index (BMI; kg/m^2^), waist circumference (WC; cm), waist-to-hip ratio (WHR), fat mass index (FMI; kg/m^2^), and fat-free mass index (FFMI; kg/m^2^). Weight, height, FMI, and FFMI were obtained from the bioelectrical impedance analysis (BIA) assessment performed using the SECA 514 mBCA analyzer (Seca GmbH & Co. KG, Hamburg, Germany), [43]. BMI was calculated as weight (kg) divided by height squared (m^2^). FMI was calculated as total fat mass divided by height squared and reflects adiposity independent of stature, whereas FFMI was calculated as total fat-free mass divided by height squared and reflects the amount of non-fat tissue, including skeletal muscle, bone, organs, and body water, relative to height. Waist circumference and hip circumference were measured using standardized anthropometric procedures, and WHR was calculated as waist circumference divided by hip circumference.

Venous blood samples were collected during clinic visits and analyzed at Hamad Medical Corporation laboratories, with results subsequently provided to QBB [42]. For this analysis, we focused on three glycemic control markers, including glycated hemoglobin A1c (HbA1c), fasting blood glucose (FBG), and fasting insulin, and four lipid profile measures, including low-density lipoprotein cholesterol (LDL-c), high-density lipoprotein cholesterol (HDL-c), total cholesterol (TC), and triacylglycerol (TG).

Systolic blood pressure (SBP) and diastolic blood pressure (DBP) were measured in the seated position using an Omron 705IT automated sphygmomanometer (Omron Corporation, Kyoto, Japan). Two readings were taken five minutes apart, and if the difference between measurements was ≥5 mmHg, a third reading was obtained. The mean of the final readings was used in the analyses [43].

### 2.3. Assessment of Dietary Data

Dietary intake was measured using a self-administered computerized food frequency questionnaire (FFQ) developed explicitly for the Qatar Biobank and adapted from the European Prospective Investigation into Cancer and Nutrition (EPIC) study [44]. The FFQ captured information on 102 food and beverage items, including consumption frequency and reported changes in dietary habits over the past year [43]. This FFQ has been used in previous population-based studies conducted within the same cohort [45,46]. A complete list of the QBB FFQ is available in Supplementary Table S1.

The Prudent dietary pattern approach used in this study has been previously applied and published in studies utilizing Qatar Biobank data in Qatari cohorts to examine their dietary intake [25,45,47]. For dietary pattern analysis, individual food items from the FFQ were grouped into thirty-eight food groups based on similarities in nutrient composition and cooking methods [47]. The weekly frequency of intake (times/week) for these food groups was entered into factor analysis to identify the Prudent dietary pattern. The number of retained factors was determined using eigenvalues greater than 1, visual inspection of the scree plot, and interpretability within the context of Qatari dietary habits. Varimax rotation was applied to improve factor interpretability. Food groups with an absolute factor loading ≥ 0.30 were considered to contribute meaningfully to the Prudent dietary pattern. The rotated factor loadings for all 38 food groups are presented in Table S2.

Individual Prudent dietary pattern scores were calculated for each participant based on the factor loadings of the Prudent dietary pattern. The distribution of Prudent dietary pattern scores is presented in Supplementary Figure S1. Because the factor scores were highly concentrated around zero, participants were ranked according to their Prudent dietary pattern scores and categorized into lower (<50th percentile) and higher (≥50th percentile) adherence groups for regression analyses.

### 2.4. Statistical Analysis

All statistical analyses were performed using Stata/SE 19 for Windows (StataCorp LLC, College Station, TX, USA). Descriptive characteristics of the study population were presented as means and standard deviations (SD) for continuous variables, and as frequencies and percentages (%) for categorical variables. Group comparisons were conducted using independent samples *t*-tests for continuous variables and Chi-square tests for categorical variables. The Shapiro–Wilk test was employed to assess the normality of all continuous variables, including the evaluation of skewness and kurtosis. Continuous outcomes with skewed distributions (BMI, WC, FFMI, FMI, FBG, fasting insulin, HbA1c, HDL-c, LDL-c, TC, TG, SBP, and DBP) were log-transformed prior to modeling. For interpretability, β coefficients were back-transformed and are presented as percent differences; where raw units are reported, models were additionally run on untransformed outcomes as a sensitivity analysis.

Dietary intake was assessed using factor scores derived from principal component analysis, which represent the intake of the Prudent diet pattern. Unlike conventional analyses that treat dietary intake as a continuous variable, the factor scores in this study were extracted and categorized into discrete values ranging from −3 to 7. For analytical purposes, factor scores for the dietary patterns were ranked and then divided into two equal-sized groups to create a binary categorical variable representing “Low” (*n* = 3595) and “High” (*n* = 3596) intake. This approach ensured an approximately equal distribution of participants across both categories, avoiding the imbalance that would have resulted from using a strict median cut-off, given the high frequency of participants with a score of zero.

Generalized linear models (GLMs) with a Gaussian family and identity link function were employed to assess the association between the Prudent dietary patterns and cardiometabolic-related outcomes such as BMI, WC, WHR, FFMI, FMI, LDL-c, HDL-c, TG, TC, SBP, and DBP. Models were adjusted for age, sex, physical activity, and BMI or WC if BMI was the outcome variable. Interaction effects were evaluated using post-estimation Wald tests. The level of significance for the association (*p*) terms was set at ≤0.05, with 95% confidence intervals estimated based on the standard errors from the fitted models. The Bonferroni correction was applied to adjust for multiple testing (*p* ≤ 0.0036), based on a significance level of *p* ≤ 0.05 divided by 14 outcome variables.

## 3. Results

### 3.1. Study Population

In this study, a total of 6919 metabolically healthy participants were included, comprising 43.5% men (*n* = 3006) and 56.5% women (*n* = 3913) (Table 1). The mean age was comparable between men and women. Anthropometric indicators revealed marked sex differences, with women having a small but significantly higher BMI and higher FMI compared to men. Conversely, men had significantly greater WC, WHR and FFMI, consistent with greater lean mass. With respect to glycemic markers, women exhibited slightly lower FBG, fasting insulin, and HbA1c levels compared with men. Lipid profiles also differed significantly between sexes. Women had more favorable lipid measures, with higher HDL-c and lower LDL-c, TG, and TC compared to men. Blood pressure was consistently higher among men. Both systolic and diastolic blood pressure were significantly elevated in men relative to women. In terms of dietary patterns, only 22.8% of the total population were classified as having high adherence to the Prudent diet (23.5% of men and 22.3% of women), indicating that fewer than one in four participants followed a diet consistent with healthier eating habits. Physical activity levels differed significantly by sex (*p* < 0.0001), with males reporting higher levels of physical activity than females. Therefore, additional analyses adjusting for physical activity were performed to account for its potential confounding effect. Overall, these findings demonstrate clear sex-specific differences across anthropometric, glycemic, lipid, and cardiovascular markers, with women showing higher adiposity measures yet more favorable lipid profiles and lower blood pressure and physical activity compared to men.

### 3.2. Association of Prudent Diet Patterns with Cardiometabolic Indicators

#### 3.2.1. Association of the Prudent Dietary Pattern with Obesity Indicators

**Figure 2:**
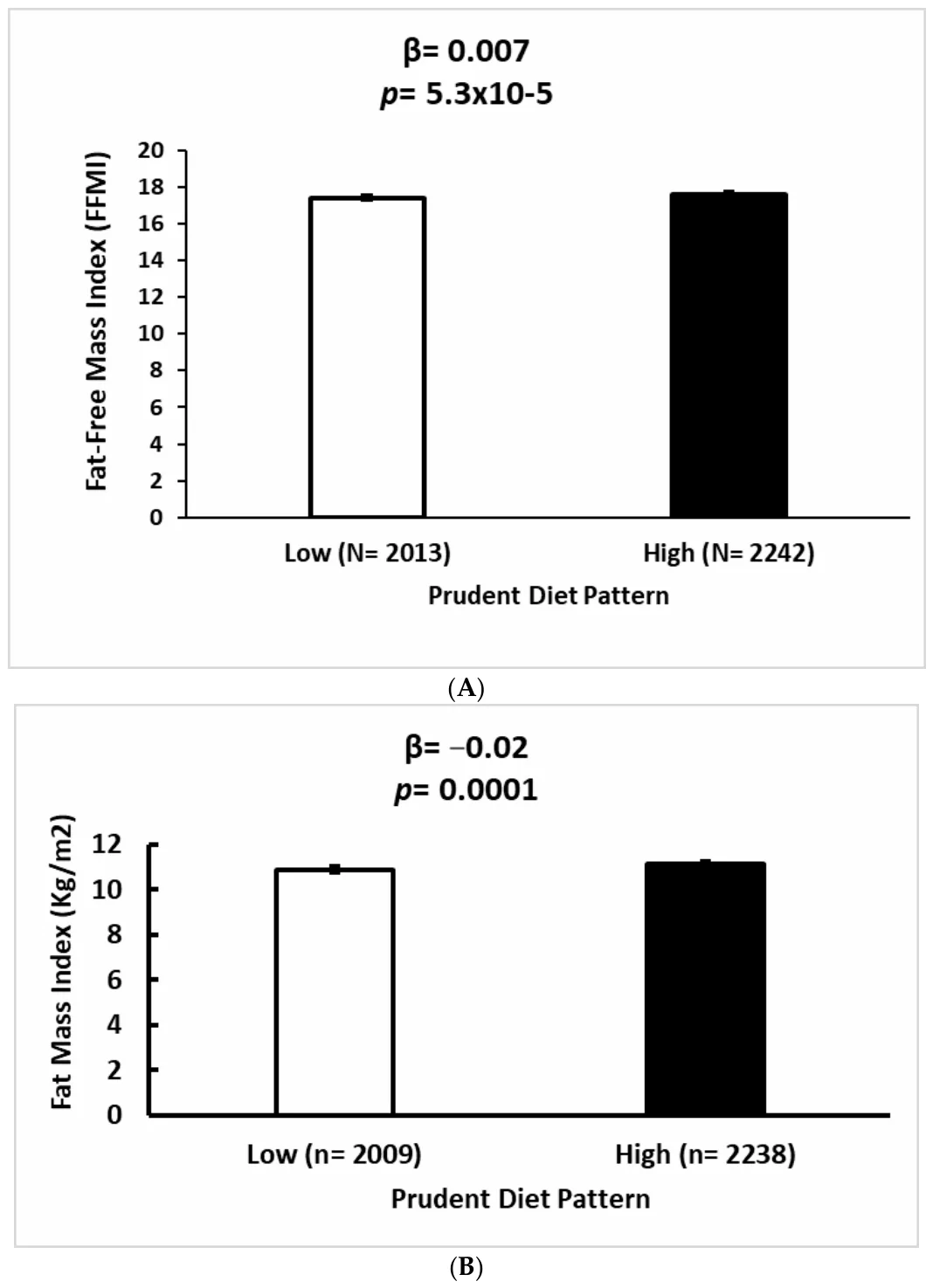
(**A**) Association between Prudent dietary pattern and fat-free mass index (FFMI). Mean FFMI values are shown for participants with low (*n* = 2013) and high (*n* = 2242) adherence to the Prudent dietary pattern. Error bars represent standard errors (SE). Generalized linear models were adjusted for age, sex, BMI, and physical activity level. The association was statistically significant (β = 0.01, *p* = 5.7 × 10^−5^), indicating that greater adherence to the Prudent dietary pattern was associated with higher fat-free mass, reflecting a more favorable body composition profile. (**B**) Association between Prudent dietary pattern and Fat mass index (FMI). Mean FMI values are shown for participants with low (*n* = 2009) and high (*n* = 2238) adherence to the Prudent dietary pattern. Error bars represent SE. Models were adjusted for age, sex, BMI and physical activity level. The association was statistically significant (β = −0.02, *p* = 0.0001), indicating lower fat mass index among individuals with greater adherence to the Prudent dietary pattern.

Analysis of the associations between the Prudent dietary pattern and cardiometabolic outcomes revealed mixed findings. No significant associations were observed for BMI, WHR, or WC as indicated by *p*-values > 0.05 (Table 2). In contrast, several body composition measures showed significant relationships. A higher Prudent diet intake was positively associated with FFMI (β = 0.007, *p* = 5.3 × 10^−5^) (Table 2, Figure 2A) and inversely associated with FMI (β = −0.02, *p* = 0.0001) (Table 2, Figure 2B). Both associations of the Prudent diet pattern with FFMI and FMI sustained significance after adjusting for Bonferroni correction.

#### 3.2.2. Association of Prudent Dietary Pattern with Type 2 Diabetes Indicators

**Figure 3:**
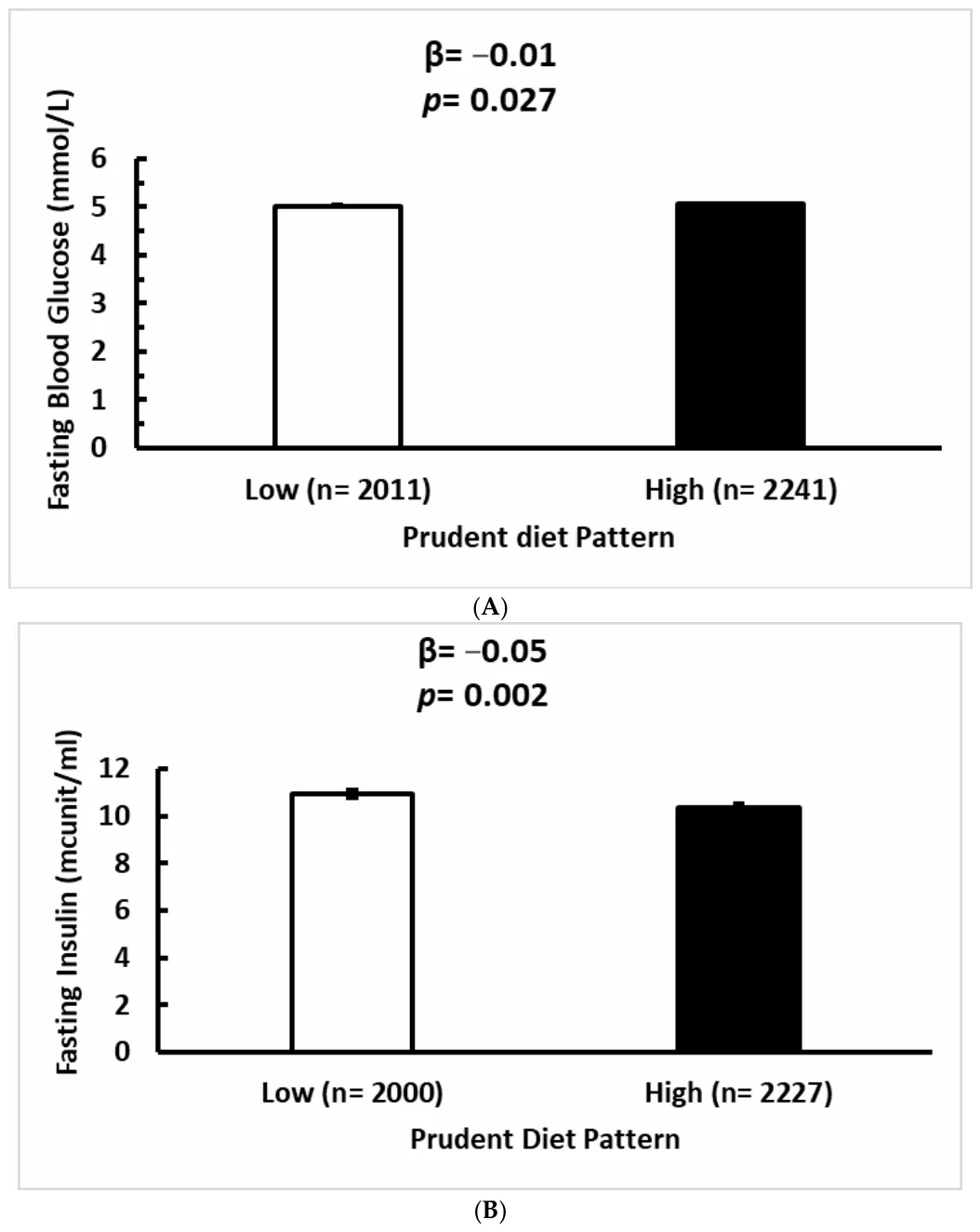
(**A**) Association of Prudent dietary pattern with fasting blood glucose (FBG). Mean fasting blood glucose values are shown for participants with low (*n* = 2000) and high (*n* = 2227) adherence to the Prudent dietary pattern. Error bars represent SE. Models were adjusted for age, sex, BMI, and physical activity level. The association was statistically significant (β = −0.01, *p* = 0.027). (**B**) Association of Prudent dietary pattern with fasting insulin levels. Mean fasting insulin values are shown for participants with low (*n* = 2011) and high (*n* = 2241) adherence to the Prudent dietary pattern. Error bars represent SE. Models were adjusted for age, sex, BMI and physical activity level. The association was statistically significant (β = −0.05, *p* = 0.0021), indicating that greater adherence to the Prudent dietary pattern was associated with lower fasting insulin levels, a marker of improved insulin sensitivity.

For glycemic outcomes, adherence to the Prudent dietary pattern showed selective but notable associations. Fasting blood glucose was significantly lower among individuals with higher adherence (β = −0.01, *p* = 0.027) (Figure 3A), and a similar inverse association was observed for fasting insulin (β = −0.05, *p* = 0.0021), indicating improved insulin sensitivity (Table 2, Figure 3B). However, only the fasting insulin association with the Prudent diet *p*-value remained significant after correction for multiple testing.

#### 3.2.3. Association of Prudent Dietary Pattern with Cardiovascular Disease Indicators

**Figure 4:**
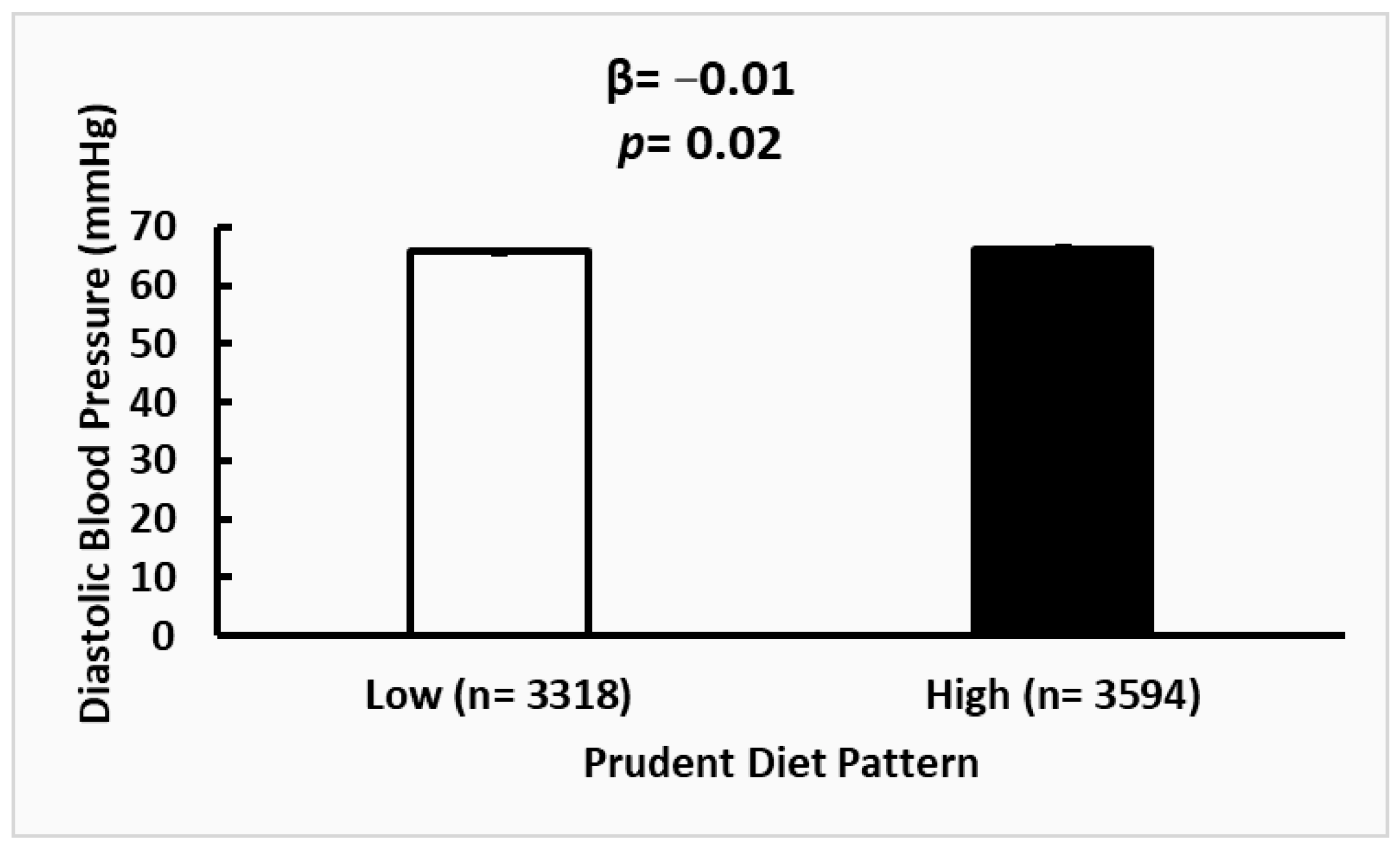
Association between the Prudent pattern and diastolic blood pressure (DBP). Mean DBP values are shown for participants with low (*n* = 3318) and high (*n* = 3594) adherence to the Prudent dietary pattern. Error bars represent SE. Models were adjusted for age, sex, BMI, and physical activity level. The association was statistically significant (β = −0.01, *p* = 0.02), suggesting lower diastolic blood pressure among participants with greater adherence to the Prudent dietary pattern.

No significant associations were observed between the Prudent dietary pattern and most lipid parameters, including HDL-c, LDL-c, TC, and TG (all *p* > 0.05) (Table 2. Similarly, systolic blood pressure showed no significant relationship with diet adherence (*p* = 0.315). In contrast, a statistically significant inverse association was detected for diastolic blood pressure (β = −0.01, *p* = 0.020) (Figure 4), suggesting that greater adherence to the Prudent dietary pattern may contribute to lower diastolic pressure (Table 2). This association did not remain significant after correction for multiple comparisons.

## 4. Discussion

In this study, greater adherence to the Prudent dietary pattern was associated with favorable cardiometabolic outcomes in a large cohort of metabolically healthy Qatari adults. Specifically, higher adherence was associated with lower FMI and fasting insulin levels, together with higher FFMI, even after Bonferroni correction. While no significant associations were observed for BMI or waist-based measures, the associations with FMI and FFMI suggest that body composition measures may be more sensitive indicators of diet-related metabolic differences than conventional anthropometric measures. These findings provide one of the first large-scale confirmations in a Qatari population that the nationally recommended dietary guidelines, which emphasize fruits, vegetables, whole grains, and limited intake of saturated fat and processed foods, are associated with more favorable metabolic profiles, particularly given that only one in four participants reported high intake of the Prudent diet pattern. This limited prevalence highlights the potential value of initiatives aimed at promoting greater adherence, which could strengthen prevention efforts against cardiometabolic diseases in the region. By demonstrating these associations in a healthy cohort, this study adds to the existing evidence supporting these dietary recommendations and provides a foundation for future intervention trials to evaluate their long-term impact across broader segments of the population.

Consistent with our findings, robust evidence demonstrates that adherence to the Prudent dietary pattern leads to significant improvements in cardiometabolic indicators across diverse populations. In a randomized controlled trial conducted among 84 healthy Canadian adults, a two-week adherence to a Prudent dietary pattern led to reductions in body fat, TC, and both SBP and DBP when compared with participants following a Western dietary pattern [48]. Higher adherence to the Prudent dietary pattern has also been associated with substantial reductions in mortality risk, with a 28% lower risk of cardiovascular mortality and a 17% lower risk of all-cause mortality reported in a large prospective cohort study of American women (*n* = 27,113) [36]. Additionally, in 281 non-Hispanic white women, adherence to this dietary pattern was inversely associated with body fat and BMI, indicating a possible protective role against adiposity [37]. Likewise, higher adherence to this pattern was inversely associated with the prevalence of obesity among Colombian adults [38]. In a cohort of 2760 Australian adults, adherence to a Prudent dietary pattern at age 21 predicted lower risks of metabolic syndrome and insulin resistance by age 30, whereas a Western dietary pattern was associated with increased risk [39]. Likewise, 1018 Irish adults following the Prudent diet pattern had lower HOMA scores and insulin resistance compared to those following a traditional diet pattern [40]. Moreover, among 6849 Chinese adults, the Prudent diet pattern was significantly associated with a 2.30 mmHg lower SBP and a 1.44 mmHg lower DBP [41]. Furthermore, in Brazilian adults, a “Healthy” dietary pattern was associated with a lower occurrence of abdominal obesity, dyslipidemia, diabetes, and hypertension in general [49]. Among Arab Lebanese adults (*n* = 305), those in the third tertile of a healthy dietary pattern score had 2.33-fold higher odds of being metabolically healthy overweight or obese compared with individuals in the first tertile [50]. Collectively, these findings support the conclusion that the Prudent diet is associated with lower adiposity, insulin resistance, and key cardiometabolic markers, including blood pressure and greater lipid regulation, across multiple populations. In addition, evidence also showed that healthy dietary patterns such as DASH, Mediterranean, and Portfolio diets are linked to significant cardiometabolic benefits, including lower cardiovascular event rates, reductions in blood pressure, and improvements in lipid profiles and inflammatory markers [51,52,53,54,55]. These findings reinforce the notion that adherence to structured healthy diets, including the Prudent pattern, can play an important role in reducing cardiometabolic risk.

The observed improvements in FMI, FFMI, fasting insulin, and blood pressure may be explained by multiple complementary pathways associated with the Prudent diet intake characterized by lean protein, vegetables, fruits, and whole grains [33,56]. Whole grain consumption has been associated with lower fasting glucose and insulin, HbA1c, and HOMA-IR compared with non-whole grain intake [57]. A key proposed mediator of these associations is the production of short-chain fatty acids (SCFAs), including acetate, propionate, and butyrate, generated by gut microbiota during the fermentation of indigestible dietary components in whole grains [58]. SCFAs enhance insulin sensitivity by acting directly on skeletal muscle and the liver to improve substrate metabolism and by activating free fatty acid receptors 2 and 3 (FFAR2/3) on enteroendocrine L-cells to stimulate the secretion of glucagon-like peptide-1 (GLP-1) and peptide YY (PYY), thereby lowering insulin demand and promoting satiety [59,60]. In addition, acetate crosses the blood–brain barrier to regulate appetite through hypothalamic signaling [61], while both acetate and propionate inhibit intracellular lipolysis via FFAR2/3, improving adipose tissue fat-buffering capacity and reducing ectopic fat deposition [62]. Butyrate may provide anti-inflammatory benefits, enhancing gut barrier function, and influencing gene regulation via epigenetic mechanisms [63]. Collectively, these SCFA-mediated actions provide one biological explanation for lower fasting insulin, reduced FMI, and higher FFMI with greater Prudent diet intake. Additionally, high fruit and vegetable intake supports vascular health by providing potassium, which lowers blood pressure through improved sodium handling and endothelial function [64]. Their vitamins and polyphenols can reduce oxidative stress, preserve nitric oxide (NO) availability, inhibit endothelin-1, and lower systemic inflammation, thereby promoting vasodilation and vascular protection [65,66]. Substituting saturated fats with unsaturated sources such as olive oil, nuts, and fish improves lipoprotein profiles and vascular outcomes [67,68]. Flavonoid-rich foods can further enhance endothelial function by stimulating endothelial nitric oxide synthase (eNOS) activity and suppressing nicotinamide adenine dinucleotide phosphate (NADPH) oxidase-related oxidative stress and inflammation, leading to improved flow-mediated dilation and modest blood pressure reductions [69]. Together, these mechanisms provide a coherent biological basis for the improvements in insulin sensitivity, body composition, and blood pressure observed with higher adherence to the Prudent diet.

This study has several notable strengths. First, it is based on a large, well-characterized cohort of 6919 metabolically healthy Qatari adults, which provided sufficient power to detect associations. Second, rigorous exclusion criteria were applied to remove participants with existing metabolic conditions, non-fasting blood samples, or pregnancy, thereby minimizing the confounding effect of disease status. Third, a wide range of cardiometabolic markers were assessed and included in this analysis, such as detailed anthropometric and biochemical measurements that move beyond reliance on BMI alone. Fourth, all phenotypic and biochemical assessments were conducted following standardized and verified protocols, ensuring the reliability of the data. Fifth, the study applied a dietary pattern approach rather than focusing on single nutrients, which provides a more comprehensive reflection of overall diet quality and captures the synergistic effects of foods consumed together. Finally, a robust statistical approach was employed, with adjustments for major confounders and the application of the Bonferroni correction to account for multiple testing, which strengthened the validity of the findings. Nevertheless, some limitations are acknowledged. The cross-sectional design precludes causal inference, as exposure and outcome were assessed at the same time point. Therefore, the temporal direction of the observed associations cannot be established, and reverse causation cannot be ruled out. A potential limitation of this study is the exclusion of non-fasting participants, which may have introduced selection bias if excluded individuals differed systematically from those included in the analysis. Dietary intake was assessed using a self-administered FFQ, which has not been formally validated in the Qatari population and is subject to recall bias. In addition, the one-year recall period may have introduced measurement error and some degree of dietary misclassification. Finally, the absence of longitudinal follow-up data limits the ability to determine whether adherence to the Prudent dietary pattern leads to sustained improvements in insulin sensitivity, body composition, and blood pressure regulation over time.

## 5. Conclusions

This study provides novel evidence from a large Qatari cohort that adherence to a Prudent dietary pattern is linked with key markers of cardiometabolic health, independent of existing disease. By demonstrating associations with fat distribution, insulin regulation, and blood pressure, our findings highlight the importance of using sensitive metabolic markers rather than relying solely on BMI. Viewed within a broader etiological framework, cardiometabolic diseases are multifactorial in nature, arising from complex interactions between genetic susceptibility and environmental exposures, among which diet represents a key, modifiable component that may both exacerbate and attenuate disease risk. These results also extend the limited evidence from Middle Eastern populations on diet–disease relationships. From a public health perspective, our findings emphasize the potential of culturally relevant dietary interventions to help address the growing burden of obesity and cardiometabolic disease in the region. While recruiting a healthy Qatari cohort enhances internal validity, the generalizability of these findings may be limited to similar Middle Eastern populations with comparable genetic backgrounds, dietary habits, and lifestyle contexts. Future longitudinal and interventional studies are needed to establish causality and to guide the integration of Prudent dietary practices into preventive strategies tailored for the Qatari population.

## Figures and Tables

**Table 1:** Characteristics of the study population.

Variables	Men (*n* = 3006) 43.5%	Women (3913) 56.5%	*p*-Value
Age (yrs)	34.8 ± 10.8	35.5 ± 11.1	0.361
BMI (kg/m^2^)	28.0 ± 5.7	28.5 ± 6.4	<0.001
WC (cm)	90.7 ± 13.5	81.1 ± 12.8	<0.0001
WHR	0.9 ± 0.1	0.8 ± 0.1	<0.0001
FMI (Kg/m^2^)	8.8 ± 4.3	12.4 ± 4.8	<0.0001
FFMI (kg/m^2^)	19.3 ± 2.0	16.4 ± 1.9	<0.001
FBG (mmol/L)	5.1 ± 1.0	5.5 ± 0.8	<0.0001
Fasting insulin (µIU/mL)	11.0 ± 8.2	10.4 ± 6.1	0.0029
HbA1c (%)	5.4 ± 0.7	5.3 ± 0.6	<0.0001
HDL-c (mmol/L)	1.2 ± 0.4	1.6 ± 0.5	<0.0001
LDL-c (mmol/L)	3.0 ± 0.9	2.7 ± 0.8	<0.0001
TC (mmol/L)	4.9 ± 1.0	4.8 ± 0.9	<0.001
TG (mmol/L)	1.4 ± 0.8	1.2 ± 0.5	<0.0001
SBP (mmHg)	113 ± 11	107 ± 12	<0.0001
DBP (mmHg)	69 ± 10	64 ± 9	<0.0001
Prudent Diet Pattern	Low = 705 (23.5%) High = 2301 (76.6%)	Low = 874 (22.3%) High = 3039 (77.7%)	0.361 *
Physical Activity	Low activity = 1534 (51%) Moderately active = 933 (31%) Vigorously active = 539 (18%)	Low activity = 2.465 (63%) Moderately active = 1017 (26%) Vigorously active = 431 (11%)	<0.0001 *

BMI: Body Mass Index; WC: Waist Circumference; WHR: Waist-to-Hip Ratio; FMI: Fat Mass Index; FFMI: Fat-Free Mass Index; FBG: Fasting Blood Glucose; HbA1c: Hemoglobin A1c; HDL-c: High-Density Lipoprotein cholesterol; LDL-c: Low-Density Lipoprotein cholesterol; TC: Total Cholesterol; TG: Triacylglycerol; SBP: Systolic Blood Pressure; DBP: Diastolic Blood Pressure; *p* value: results from independent *t*-test; *: results from Chi-square test.

**Table 2:** Association of the Prudent diet pattern with indicators of obesity, type 2 diabetes and cardiovascular disease.

Outcomes	*p*-Value	*β* Coefficient
BMI (kg/m^2^)	0.061	0.0291
WHR	0.378	0.0014
WC (cm)	0.857	0.0003
FFMI (Kg/m^2^)	5.3 × 10^−5^ *	0.007
FMI (Kg/m^2^)	0.0001 *	−0.020
FBG (mmol/L)	**0.027**	−0.0096
Fasting insulin (µIU/mL)	0.002 *	−0.0463
HbA1c (%)	0.747	−0.0008
HDL-c (mmol/L)	0.168	−0.0108
LDL-c (mmol/L)	0.1141	0.0113
TC (mmol/L)	0.138	0.0066
TG (mmol/L)	0.0612	0.0159
SBP (mmHg)	0.2817	0.0024
DBP (mmHg)	**0.0204**	−0.0077

BMI: Body Mass Index; WHR: Waist-to-Hip Ratio; WC: Waist Circumference; FFMI: Fat-Free Mass Index; FMI: Fat Mass Index; FBG: Fasting Blood Glucose; HbA1c: Hemoglobin A1c; HDL-c: High-Density Lipoprotein cholesterol; LDL-c: Low-Density Lipoprotein cholesterol; TG: Triacylglycerol; TC: Total Cholesterol; SBP: Systolic Blood Pressure; DBP: Diastolic Blood Pressure. *p* value: adjusted for age, sex, BMI and physical activity level; **Bold**: Significant *p*-value; *: Significant after Bonferroni correction.

## Data Availability

All relevant data are contained within the paper and its Supporting Information Files.

## Supplementary Materials

The following supporting information can be downloaded at https://www.mdpi.com/article/10.3390/nu18162607/s1: Table S1, Food items included in the Qatar Biobank 102-item Food Frequency Questionnaire (FFQ); Table S2, Rotated factor loadings for the Prudent diet pattern across the 38 food groups; Figure S1, Prudent diet factor scores of study participants (*n* = 7191).

## Abbreviations

The following abbreviations are used in this manuscript:

QBB	Qatar Biobank
CMD	Cardiometabolic Disease
T2D	Type-2 diabetes
CVD	Cardiovascular disease
BMI	Body mass index
WC	Waist circumference
WHR	Waist-to-Hip Ratio
FFMI	Fat-free mass index
FMI	Fat mass index
FBG	Fasting Blood Glucose
LDL-c	High-Density Lipoprotein cholesterol
HDL-c	Low-Density Lipoprotein cholesterol
TG	Triacylglycerol
TC	Total Cholesterol
SBP	Systolic Blood Pressure
DBP	Diastolic Blood Pressure
