# Supplementary material for: Association Between Prudent Dietary Pattern and Cardiometabolic Indicators in a Healthy Qatari Population: Evidence from the Qatar Biobank (QBB)
Source: Nutrients. 2026 Aug 10;18(16):2607. doi: 10.3390/nu18162607 (PMC13516244; doi:10.3390/nu18162607)
Supplement: Supplementary file 1 [file nutrients-18-02607-s001.zip › nutrients-4414245-supplementary.pdf]

Table S1: Food items included in the Qatar Biobank 102-item Food Frequency Questionnaire (FFQ)

**Have you modified your diet in the last year?**
• No
• Yes
• Prefer Not To Answer
**Why did you modify your diet? (You can select more than one answer)**
• None of the above
• Prefer Not To Answer
• To lose weight
• Because of hypertension (raised blood pressure)
• Because of diabetes
• Because of high cholesterol
• Because of another disease
• Because of loss of appetite
• To have a healthier diet
**Do you have a special diet?**
• Prefer Not To Answer
• No, I don't have a special diet
• Low calorie diet
• Low salt diet
• Low fat diet
• Vegan diet (no meat, fish, eggs, dairy products)
• Vegetarian diet (allows dairy products)
• I do not eat red meat
• Other special diet

**How often during a typical week in the last year did you eat from a common plate, shared with others?**
• In general, every day
• Not every day but more than 3 times in a week
• 1-3 times per week
• Not every week but at least once in a month
• Never or rarely
• Prefer Not To Answer
• Less than once per week
• Once or twice per week
• 3-5 times a week
• Every day or almost everyday
• Never or rarely
• Prefer Not To Answer
**How often during a typical week in the last year did you eat snacks between meals? (Meals: breakfast, lunch and dinner)**
• Never or rarely
• More than 7 times per week
• 6-7 times per week
• Once or twice per week
• 3-5 times a week
• Prefer Not To Answer
**Select the option that best describes how often you drank coffee or tea during the last year. Please ensure one response entered per row.**
• Prefer Not To Answer
**Select the option that best describes how often you drank Arabic_coffee during the last year.**
• Never or rarely
• 1-3 times per month

• 1-3 times per week
• 4-6 times a week
• Once per day
• Twice per day
• 3 or more times per day
**Select the option that best describes how often you drank Instant coffee during the last year.**
• Never or rarely
• 1-3 times per month
• 1-3 times per week
• 4-6 times a week
• Once per day
• Twice per day
• 3 or more times per day
**Select the option that best describes how often you drank Other coffee (eg filter coffee, cappuccino) during the last year.**
• Never or rarely
• 1-3 times per month
• 1-3 times per week
• 4-6 times a week
• Once per day
• Twice per day
• 3 or more times per day
**Select the option that best describes how often you drank Tea during the last year.**
• Never or rarely
• 1-3 times per month
• 1-3 times per week

• 4-6 times a week
• Once per day
• Twice per day
• 3 or more times per day
**Select the option that best describes how often you drank Herbal_tea during the last year.**
• Never or rarely
• 1-3 times per month
• 1-3 times per week
• 4-6 times a week
• Once per day
• Twice per day
• 3 or more times per day
**Select the option that best describes how often you drank Karak during the last year.**
• Never or rarely
• 1-3 times per month
• 1-3 times per week
• 4-6 times a week
• Once per day
• Twice per day
• 3 or more times per day
**How many teaspoons of sugar did you most often put in your coffee or tea during the last year? Please ensure one response entered per row.**
• Prefer Not To Answer
**Sugar in instant coffee or non- Arabic coffee**
• None
• 1

• 2
• 3
• >3
• I don't drink tea/coffee
**Sugar in tea or herbal tea**
• None
• 1
• 2
• 3
• >3
• I don't drink tea/coffee
**For each item listed below, select the option that best describes how often you ate or drank those items during the last year. Please ensure one response entered per row.**
• Prefer Not To Answer
**Milk as a beverage (cold milk, cappuccino)**
• Never or rarely
• 1-3 times per month
• 1-3 times per week
• 4-6 times a week
• Once per day
• 2 or more times per day
**Milk added to cereals**
• Never or rarely
• 1-3 times per month
• 1-3 times per week
• 4-6 times a week

• Once per day
• 2 or more times per day
**Milk shakes or flavoured milk**
• Never or rarely
• 1-3 times per month
• 1-3 times per week
• 4-6 times a week
• Once per day
• 2 or more times per day
**Laban**
• Never or rarely
• 1-3 times per month
• 1-3 times per week
• 4-6 times a week
• Once per day
• 2 or more times per day
**Yoghurt**
• Never or rarely
• 1-3 times per month
• 1-3 times per week
• 4-6 times a week
• Once per day
• 2 or more times per day
**Labneh**
• Never or rarely
• 1-3 times per month

• 1-3 times per week
• 4-6 times a week
• Once per day
• 2 or more times per day
**Other white cheese (halloumi, cream cheese)**
• Never or rarely
• 1-3 times per month
• 1-3 times per week
• 4-6 times a week
• Once per day
• 2 or more times per day
**Processed cheese**
• Never or rarely
• 1-3 times per month
• 1-3 times per week
• 4-6 times a week
• Once per day
• 2 or more times per day
**Hard cheese (such as gouda, cheddar)**
• Never or rarely
• 1-3 times per month
• 1-3 times per week
• 4-6 times a week
• Once per day
• 2 or more times per day

**Butter**
• Never or rarely
• 1-3 times per month
• 1-3 times per week
• 4-6 times a week
• Once per day
• 2 or more times per day
**Select the option that best describes what type of milk you consumed most often during the last year.**
• Whole fat milk
• Reduced fat milk
• Fat free milk
• All types of milk equally
• Other; eg Soya, almond etc
• I don't know
• Prefer Not To Answer
**FAST FOODS. For each item listed below, select the option that best describes how often you ate those items during the last year. Please ensure one response entered per row.**
• Prefer Not To Answer
**Meat shawarma**
• Never or rarely
• 1-3 times per month
• 1-3 times per week
• 4-6 times a week
• Once per day
• 2 or more times per day
**Chicken shawarma**
• Never or rarely

• 1-3 times per month
• 1-3 times per week
• 4-6 times a week
• Once per day
• 2 or more times per day
**Falafel wrap**
• Never or rarely
• 1-3 times per month
• 1-3 times per week
• 4-6 times a week
• Once per day
• 2 or more times per day
**Samosa**
• Never or rarely
• 1-3 times per month
• 1-3 times per week
• 4-6 times a week
• Once per day
• 2 or more times per day
**Other middle-eastern style fast foods**
• Never or rarely
• 1-3 times per month
• 1-3 times per week
• 4-6 times a week
• Once per day
• 2 or more times per day

**French fries**
• Never or rarely
• 1-3 times per month
• 1-3 times per week
• 4-6 times a week
• Once per day
• 2 or more times per day
**Potato chips**
• Never or rarely
• 1-3 times per month
• 1-3 times per week
• 4-6 times a week
• Once per day
• 2 or more times per day
**Burgers, hotdogs**
• Never or rarely
• 1-3 times per month
• 1-3 times per week
• 4-6 times a week
• Once per day
• 2 or more times per day
**Pizza**
• Never or rarely
• 1-3 times per month
• 1-3 times per week
• 4-6 times a week

• Once per day
• 2 or more times per day
**Fried chicken, e.g. wings, nuggets**
• Never or rarely
• 1-3 times per month
• 1-3 times per week
• 4-6 times a week
• Once per day
• 2 or more times per day
**DRINKS. For each beverage listed below, select the option that best describes how often you drank those beverages during the last year. Please ensure one response entered per row.**
• Prefer Not To Answer
**Fresh fruit juices**
• Never or rarely
• 1-3 times per month
• 1-3 times per week
• 4-6 times a week
• Once per day
• 2 or more times per day
**Smoothies**
• Never or rarely
• 1-3 times per month
• 1-3 times per week
• 4-6 times a week
• Once per day
• 2 or more times per day

**Preserved fruit juice (canned/bottled)**
• Never or rarely
• 1-3 times per month
• 1-3 times per week
• 4-6 times a week
• Once per day
• 2 or more times per day
**Soft drinks, sodas**
• Never or rarely
• 1-3 times per month
• 1-3 times per week
• 4-6 times a week
• Once per day
• 2 or more times per day
**Diet soft drinks, sodas**
• Never or rarely
• 1-3 times per month
• 1-3 times per week
• 4-6 times a week
• Once per day
• 2 or more times per day
**Energy drinks**
• Never or rarely
• 1-3 times per month
• 1-3 times per week
• 4-6 times a week

• Once per day
• 2 or more times per day
**For each item listed below, select the option that best describes how often you ate those items during the last year. Do not include the bread eaten with fast foods - shawarma, kufta, hamburgers, wraps. Please ensure one response entered per row.**
• Prefer Not To Answer
**Arabic bread (Lavash)**
• Never or rarely
• 1-3 times per month
• 1-3 times per week
• 4-6 times a week
• Once per day
• 2 times per day
• 3 or more times per day
**Iranian bread**
• Never or rarely
• 1-3 times per month
• 1-3 times per week
• 4-6 times a week
• Once per day
• 2 times per day
• 3 or more times per day
**Toast (sliced bread) - white**
• Never or rarely
• 1-3 times per month
• 1-3 times per week
• 4-6 times a week

• Once per day
• 2 times per day
• 3 or more times per day
**Toast (sliced bread) - brown**
• Never or rarely
• 1-3 times per month
• 1-3 times per week
• 4-6 times a week
• Once per day
• 2 times per day
• 3 or more times per day
**Other breads**
• Never or rarely
• 1-3 times per month
• 1-3 times per week
• 4-6 times a week
• Once per day
• 2 times per day
• 3 or more times per day
**Croissant or other pastries**
• Never or rarely
• 1-3 times per month
• 1-3 times per week
• 4-6 times a week
• Once per day
• 2 times per day

• 3 or more times per day
**Zaatar fatayer, other fatayer**
• Never or rarely
• 1-3 times per month
• 1-3 times per week
• 4-6 times a week
• Once per day
• 2 times per day
• 3 or more times per day
**For each item listed below, select the option that best describes how often you ate or drank those items during the last year. Please ensure one response entered per row.**
• Prefer Not To Answer
**Balaleet**
• Never or rarely
• 1-3 times per month
• 1-3 times per week
• 4-6 times a week
• Once per day or more
**Cornflakes and other cold cereals**
• Never or rarely
• 1-3 times per month
• 1-3 times per week
• 4-6 times a week
• Once per day or more
**Muesli, porridge, other bran cereals**
• Never or rarely
• 1-3 times per month

• 1-3 times per week
• 4-6 times a week
• Once per day or more
**FISH AND EGG DISHES. For each item listed below, select the option that best describes how often you ate those items during the last year. Please ensure one response entered per row. Average frequency of consumption during the last year**
• Prefer Not To Answer
**Grilled, fried or baked fish**
• Never or rarely
• 1-3 times per month
• 1-3 times per week
• 4-6 times a week
• Everyday
**Fish cooked with vegetables**
• Never or rarely
• 1-3 times per month
• 1-3 times per week
• 4-6 times a week
• Everyday
**Smoked fish**
• Never or rarely
• 1-3 times per month
• 1-3 times per week
• 4-6 times a week
• Everyday
**Eggs (boiled, fried, omelettes, scrambled)**
• Never or rarely

• 1-3 times per month
• 1-3 times per week
• 4-6 times a week
• Everyday
**DESSERTS AND SNACKS. For each item listed below, select the option that best describes how often you ate those items during the last year. Please ensure one response entered per row. Average frequency of consumption during the last year**
• Prefer Not To Answer
**Traditional desserts (mahalabea, halwa, aquili, elgayemat, kammafaroursh)**
• Never or rarely
• Once a month
• 2-3 times per week
• 4-6 times a week
• Once per day
• 2 or more times per day
• 2-3 times a month
• Once a week
**Other Middle Eastern or Lebanese desserts**
• Never or rarely
• Once a month
• 2-3 times per week
• 4-6 times a week
• Once per day
• 2 or more times per day
• 2-3 times a month
• Once a week

**Cookies, biscuits**
• Never or rarely
• Once a month
• 2-3 times per week
• 4-6 times a week
• Once per day
• 2 or more times per day
• 2-3 times a month
• Once a week
**Muffin, cake, doughnut**
• Never or rarely
• Once a month
• 2-3 times per week
• 4-6 times a week
• Once per day
• 2 or more times per day
• 2-3 times a month
• Once a week
**Chocolate bar**
• Never or rarely
• Once a month
• 2-3 times per week
• 4-6 times a week
• Once per day
• 2 or more times per day
• 2-3 times a month

• Once a week
**Ice cream**
• Never or rarely
• Once a month
• 2-3 times per week
• 4-6 times a week
• Once per day
• 2 or more times per day
• 2-3 times a month
• Once a week
**Nuts**
• Never or rarely
• Once a month
• 2-3 times per week
• 4-6 times a week
• Once per day
• 2 or more times per day
• 2-3 times a month
• Once a week
**FRUITS. How often did you eat fruits in the last year (Do not include juices here)? Please ensure one response entered per row. Average frequency of consumption during the last year**
• Prefer Not To Answer
**Fresh fruits**
• Never or rarely
• 1-3 times per month
• 1-3 times per week

• 4-6 times a week
• Once per day
• 2 or more times per day
**Canned fruits**
• Never or rarely
• 1-3 times per month
• 1-3 times per week
• 4-6 times a week
• Once per day
• 2 or more times per day
**Dry fruits**
• Never or rarely
• 1-3 times per month
• 1-3 times per week
• 4-6 times a week
• Once per day
• 2 or more times per day
**Dates**
• Never or rarely
• 1-3 times per month
• 1-3 times per week
• 4-6 times a week
• Once per day
• 2 or more times per day
**Banana**
• Never or rarely

• 1-3 times per month
• 1-3 times per week
• 4-6 times a week
• Once per day
• 2 or more times per day
**Watermelon, sweet melon (cantaloupe)**
• Never or rarely
• 1-3 times per month
• 1-3 times per week
• 4-6 times a week
• Once per day
• 2 or more times per day
**How often did you eat meat, chicken or fish in the last year? Do not include sandwiches, wraps, shawarma, samosa, pizza, pies. Please ensure one response entered per row. Average frequency of consumption during the last year**
• Prefer Not To Answer
**Red Meat (Beef, lamb, goat, camel)**
• Never or rarely
• 1-3 times per month
• 1-3 times per week
• 4-6 times a week
• Once per day
• 2 or more times per day
**Light Meat (Chicken, duck)**
• Never or rarely
• 1-3 times per month
• 1-3 times per week

• 4-6 times a week
• Once per day
• 2 or more times per day
**Fish**
• Never or rarely
• 1-3 times per month
• 1-3 times per week
• 4-6 times a week
• Once per day
• 2 or more times per day
**CHICKEN AND MEAT DISHES. For each item listed below, select the option that best describes how often you ate those items during the last year. Please ensure one response entered per row.**
• Prefer Not To Answer
**Meat served with rice (Mashboos)**
• Never or rarely
• 1-3 times per month
• 1-3 times per week
• 4-6 times a week
• Everyday
**Chicken with rice**
• Never or rarely
• 1-3 times per month
• 1-3 times per week
• 4-6 times a week
• Everyday
**Harees**
• Never or rarely

• 1-3 times per month
• 1-3 times per week
• 4-6 times a week
• Everyday
**Meat cooked with vegetables (Margooga, Saloona)**
• Never or rarely
• 1-3 times per month
• 1-3 times per week
• 4-6 times a week
• Everyday
**Chicken cooked with vegetables (Margooga, Saloona)**
• Never or rarely
• 1-3 times per month
• 1-3 times per week
• 4-6 times a week
• Everyday
**Meat curry**
• Never or rarely
• 1-3 times per month
• 1-3 times per week
• 4-6 times a week
• Everyday
**Chicken curry**
• Never or rarely
• 1-3 times per month
• 1-3 times per week

• 4-6 times a week
• Everyday
**Kofta**
• Never or rarely
• 1-3 times per month
• 1-3 times per week
• 4-6 times a week
• Everyday
**Meat kebab**
• Never or rarely
• 1-3 times per month
• 1-3 times per week
• 4-6 times a week
• Everyday
**Chicken kebab**
• Never or rarely
• 1-3 times per month
• 1-3 times per week
• 4-6 times a week
• Everyday
**Lamb chops, escalope**
• Never or rarely
• 1-3 times per month
• 1-3 times per week
• 4-6 times a week
• Everyday

**Grilled chicken**
• Never or rarely
• 1-3 times per month
• 1-3 times per week
• 4-6 times a week
• Everyday
**For each item listed below, select the option that best describes how often you ate or drank those items during the last year. Please ensure one response entered per row.**
• Prefer Not To Answer
**Ready-made soup from powder**
• Never or rarely
• 1-3 times per month
• 1-3 times per week
• 4-6 times a week
• Once per day or more
**Other soups**
• Never or rarely
• 1-3 times per month
• 1-3 times per week
• 4-6 times a week
• Once per day or more
**Hummus, mutabal, baba ghanush, other dips**
• Never or rarely
• 1-3 times per month
• 1-3 times per week
• 4-6 times a week
• Once per day or more

**Tabbouleh**
• Never or rarely
• 1-3 times per month
• 1-3 times per week
• 4-6 times a week
• Once per day
• 2 or more times per day
**Koussa mahshi (stuffed courgettes)**
• Never or rarely
• 1-3 times per month
• 1-3 times per week
• 4-6 times a week
• Once per day or more
**Eggplants fried or baked, moussaka**
• Never or rarely
• 1-3 times per month
• 1-3 times per week
• 4-6 times a week
• Once per day or more
**Other vegetable starters**
• Never or rarely
• 1-3 times per month
• 1-3 times per week
• 4-6 times a week
• Once per day or more

**Fish and seafoods starters**
• Never or rarely
• 1-3 times per month
• 1-3 times per week
• 4-6 times a week
• Once per day or more
**Chicken, meat, or sausage starters**
• Never or rarely
• 1-3 times per month
• 1-3 times per week
• 4-6 times a week
• Once per day or more
**POTATOES, RICE, NOODLES AND PASTA. For each item listed below, select the option that best describes how often you ate those items during the last year. Please ensure one response entered per row.**
• Prefer Not To Answer
**Boiled, baked potatoes**
• Never or rarely
• 1-3 times per month
• 1-3 times per week
• 4-6 times a week
• Once per day
• 2 or more times per day
**White rice**
• Never or rarely
• 1-3 times per month
• 1-3 times per week
• 4-6 times a week

• Once per day
• 2 or more times per day
**Vegetables biryani**
• Never or rarely
• 1-3 times per month
• 1-3 times per week
• 4-6 times a week
• Once per day
• 2 or more times per day
**Chicken biryani**
• Never or rarely
• 1-3 times per month
• 1-3 times per week
• 4-6 times a week
• Once per day
• 2 or more times per day
**Asian-style noodles**
• Never or rarely
• 1-3 times per month
• 1-3 times per week
• 4-6 times a week
• Once per day
• 2 or more times per day
**Italian-style pasta, lasagna**
• Never or rarely
• 1-3 times per month

• 1-3 times per week
• 4-6 times a week
• Once per day
• 2 or more times per day
**SALADS, COOKED AND RAW VEGETABLES. For each item listed below, select the option that best describes how often you ate those items during the last year. Please ensure one response entered per row.**
• Prefer Not To Answer
**Raw vegetables**
• Never or rarely
• 1-3 times per month
• 1-3 times per week
• 4-6 times a week
• Once per day
• 2 or more times per day
**Green leafy salad (e.g. lettuce)**
• Never or rarely
• 1-3 times per month
• 1-3 times per week
• 4-6 times a week
• Once per day
• 2 or more times per day
**Mixed salad (for example with tomato, onion, cucumber, other vegetables)**
• Never or rarely
• 1-3 times per month
• 1-3 times per week
• 4-6 times a week

• Once per day
• 2 or more times per day
**Fattoush**
• Never or rarely
• 1-3 times per month
• 1-3 times per week
• 4-6 times a week
• Once per day
• 2 or more times per day
**Beans salads (chickpeas, lentils)**
• Never or rarely
• 1-3 times per month
• 1-3 times per week
• 4-6 times a week
• Once per day
• 2 or more times per day
**Bulghur salad**
• Never or rarely
• 1-3 times per month
• 1-3 times per week
• 4-6 times a week
• Once per day
• 2 or more times per day
**Vegetable stews**
• Never or rarely
• 1-3 times per month

• 1-3 times per week
• 4-6 times a week
• Once per day
• 2 or more times per day
**Vegetable curries**
• Never or rarely
• 1-3 times per month
• 1-3 times per week
• 4-6 times a week
• Once per day
• 2 or more times per day
**Other cooked vegetables**
• Never or rarely
• 1-3 times per month
• 1-3 times per week
• 4-6 times a week
• Once per day
• 2 or more times per day
**Corn**
• Never or rarely
• 1-3 times per month
• 1-3 times per week
• 4-6 times a week
• Once per day
• 2 or more times per day

Table S2. Rotated factor loadings for the Prudent diet pattern across the 38 food groups.

No.	Food Group	Prudent Dietary Pattern
1	Coffee	0.2414
2	Tea	0.2876
3	Milk	0.0574
4	Milk added to cereal	0.0760
5	Milk shakes	0.0695
6	Yogurt	0.2714
7	Cheese	0.1239
8	Butter	0.0784
9	Breakfast cereal	0.2205
10	Arabic/Iranian bread	0.2992
11	White bread	0.0403
12	Brown bread	0.0199
13	Other bread	0.1236
14	Croissant	0.1266
15	Zaatar fatayer	0.2077
16	Soups/starters	**0.5680**
17	Salad and raw vegetables	**0.7176**
18	Salad and cooked vegetables	**0.5637**
19	Potato	**0.3695**
20	White rice	**0.4032**
21	Biryani	**0.3678**
22	Asian noodle	0.2678
23	Italian-style pasta (Lasagna)	0.2063
24	Red meat	**0.3571**
25	Chicken	**0.3113**
26	Fish	**0.6295**
27	Chicken/meat fish mixed dish	**0.4896**
28	Grilled/fried/baked fish	**0.6273**
29	Eggs	**0.3875**
30	Fast food	0.2148
31	Fresh fruit	**0.7068**
32	Canned/dried fruit and dates	**0.6178**
33	Desserts	0.1674
34	Chocolate	-0.0364
35	Ice cream	0.0734
36	Nuts	**0.3835**
37	Fresh fruit juice	**0.5256**
38	Soft drink	0.0479

Food groups with factor loadings **≥0.30** (are in **bold**) were considered to contribute meaningfully to the Prudent dietary pattern and were used to characterize it. Positive factor loadings indicate a positive contribution to the dietary pattern, whereas negative factor loadings indicate an inverse association.

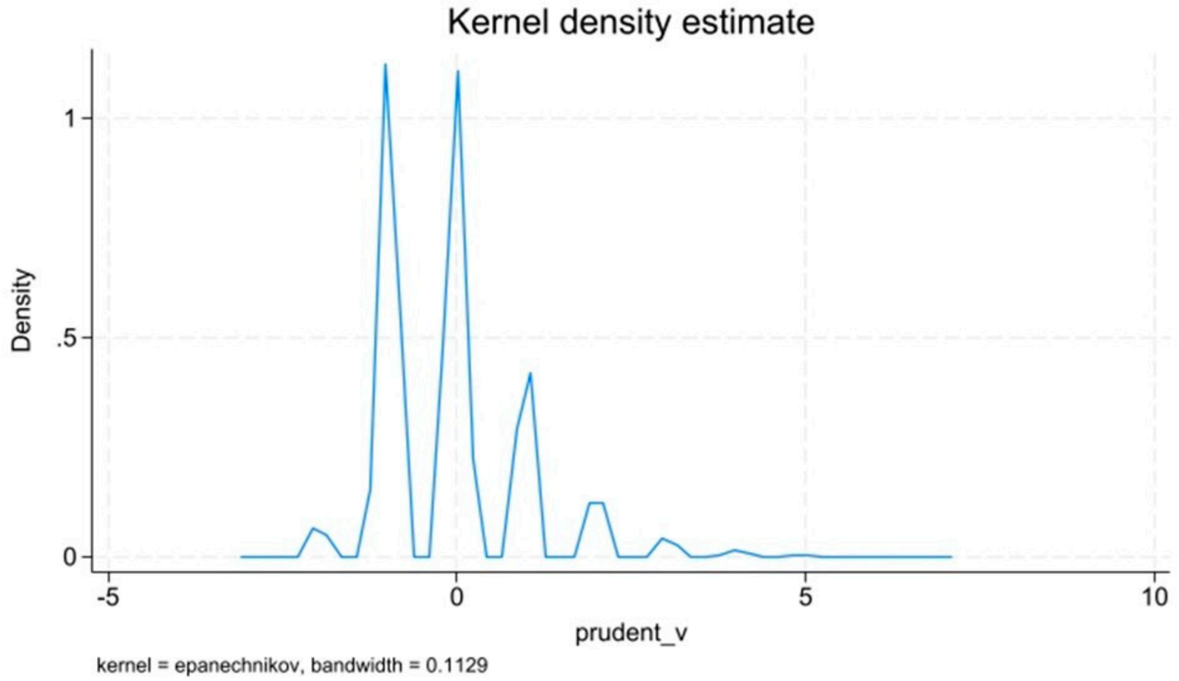

Figure S1: Prudent diet factor scores of study participants (n = 7191).
